# Supplementary material for: Estimating the heritability of SARS-CoV-2 susceptibility and COVID-19 severity
Source: Nat Commun. 2024 Jan 8;15:367. doi: 10.1038/s41467-023-44250-7 (PMC10774300; doi:10.1038/s41467-023-44250-7)
Supplement: Supplementary file 1 — Supplementary Information [file 41467_2023_44250_MOESM1_ESM.pdf]

Estimating the Heritability of SARS-CoV-2 Susceptibility and COVID-19 Severity

Kathleen LaRow Brown[1], Vijendra Ramlall[1,2], Michael Zietz[1], Undina Gisladottir[1], Nicholas P. Tatonetti[1,3,4*]

1. Department of Biomedical Informatics, Columbia University, New York, NY, USA
2. Department of Physiology & Cellular Biophysics, Columbia University, New York, NY, USA.
3. Department of Computational Biomedicine, Cedars-Sinai Medical Center, West Hollywood, CA, USA
4. Cedars-Sinai Cancer, Cedars-Sinai Medical Center, Los Angeles, CA, USA

Supplementary Methods

Permutation Analysis

To gain confidence that genetics and shared environment account for phenotypic differences between individuals we created negative controls by permuting the patient values once for days hospitalized and once for susceptibility (the two phenotypes we have quality estimates for) and repeated our analyses. We set SOLARStrap to run 200 iterations of SOLAR using between 20% and 90% of available families and included sex and age as covariates. We would expect that our models would not attribute phenotypic variance to either heritability or shared environment for these permuted phenotypes.

Supplementary Figures

Supplementary Figure 1. Stability of shared environment and heritability estimates at different sample sizes and modelling criteria

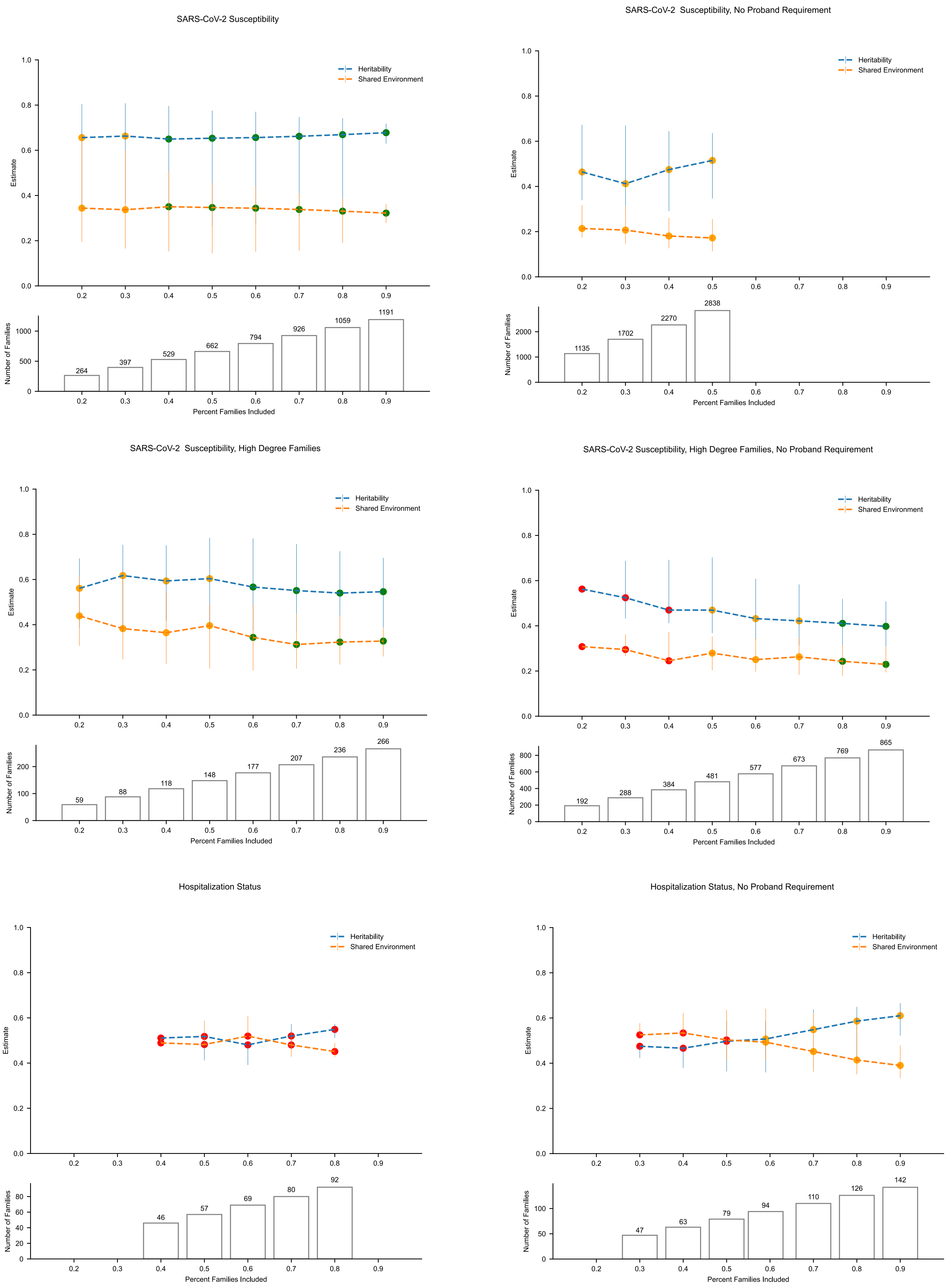

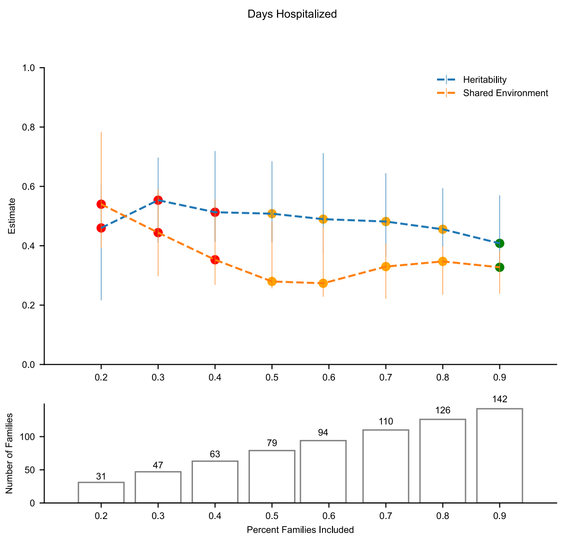

Supplementary Figure 1. Heritability and Shared Environment estimates with 95% CI at different sample size inclusion. Center points are the heritability and shared environment estimates from the model associated with the median, significant heritability estimate. Scatter plot colors denote Quality Score (see Polubriaginof et al.'s discussion of POSA)[1]. Quality Scores $\leq$ 0.2 are poor quality estimates and are colored red. Estimates with Quality Scores 0.2 < Quality Scores $\leq$ 0.8 are medium quality and are colored yellow. Quality Scores > 0.8 are high quality and are colored green. Binary traits were modeled with (default) and without a proband requirement. Families in the proband requirement iterations were required to have at least one case in the family for inclusion. Source data are provided as a Source Data file.

Supplementary Table 1. Shared environment and heritability point estimates

Category	Trait	Proband	Narrow-sense Heritability Estimate	Shared Environment Estimate	Families	Quality Score
Susceptibility	+ PCR	Yes	65% (95% CI: 33%-80%)	35% (95% CI:15%-51%)	1324	0.84
	+ PCR High Degree Families	Yes	57% (95% CI: 37%-78%)	34% (95% CI:20%-49%)	296	0.86
	+ PCR	No	52% (95% CI: 35%-64%)	17% (95% CI:11%-26%)	5676	0.8
	+ PCR High Degree Families	No	41% (95% CI: 31%-52%)	24% (95% CI:18%-31%)	962	0.82
	Reinfection	N/A	Insufficient Sample		17	
Severity	Hospitalization	Yes	55% (95% CI: 51%-57%)	45% (95% CI:43%-49%)	115	0.03
	Hospitalization	No	61% (95% CI: 52%-67%)	39% (95% CI:33%-48%)	158	0.44
	Days Hospitalized	-	41% (95% CI: 33%-57%)	33% (95% CI:24%-38%)	158	0.9
	+PCR w/ Intubation	-	Insufficient Sample		26	
	+PCR w/ Death	-	Insufficient Sample		0	

Supplementary Table 1 Highest quality Heritability and Shared Environment results as determined by the Quality Score and sample size. Quality Scores $\leq$ 0.2 are poor quality estimates. Estimates with Quality Scores 0.2 < Quality Scores $\leq$ 0.8 are medium quality and Quality Scores > 0.8 are high quality (see Polubriaginof et al.'s discussion of POSA)[1]. No estimate means no phenotypic variance was accounted for. Binary traits were modeled with and without a proband requirement.

Supplementary Figure 2. Stability of shared environment and heritability estimates at different sample sizes and modelling criteria with covariates excluded

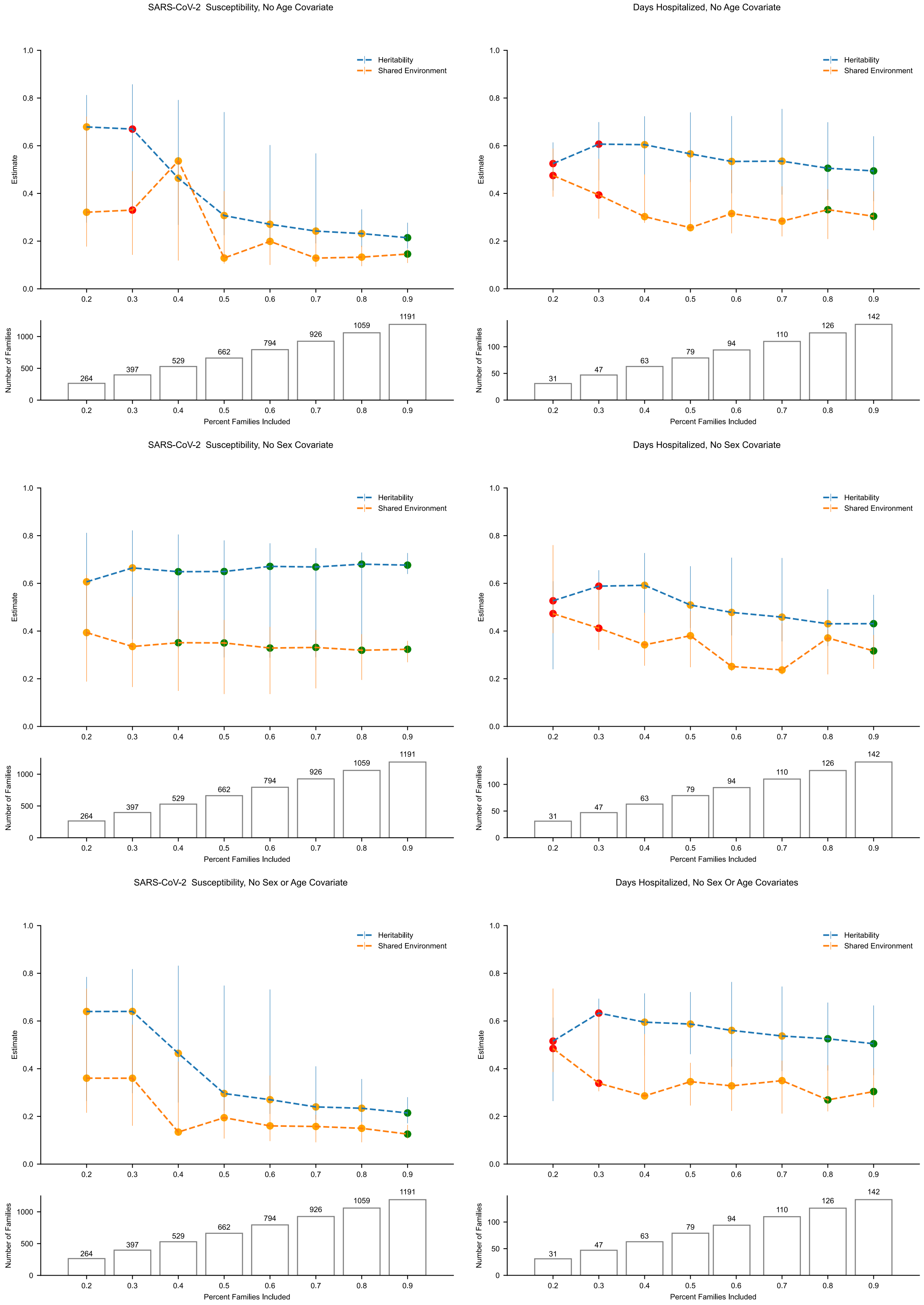

Supplementary Figure 2. Heritability and shared environment estimates with 95% CI at different sample size inclusion with age, sex, and age and sex covariates removed from the models. Center points are the heritability and shared environment estimates from the model associated with the median, significant heritability estimate. Scatter plot colors denote Quality Score (see Polubriaginof et al.'s discussion of POSA.)[1] Quality Scores $\leq$ 0.2 are poor quality estimates and are colored red. Estimates with Quality Scores 0.2 < Quality Scores $\leq$ 0.8 are medium quality and are colored yellow. Quality Scores > 0.8 are high quality and are colored green. Binary traits were modeled with proband requirement. Source data are provided as a Source Data file.

Supplementary Figure 3. Case and control group age distributions over time

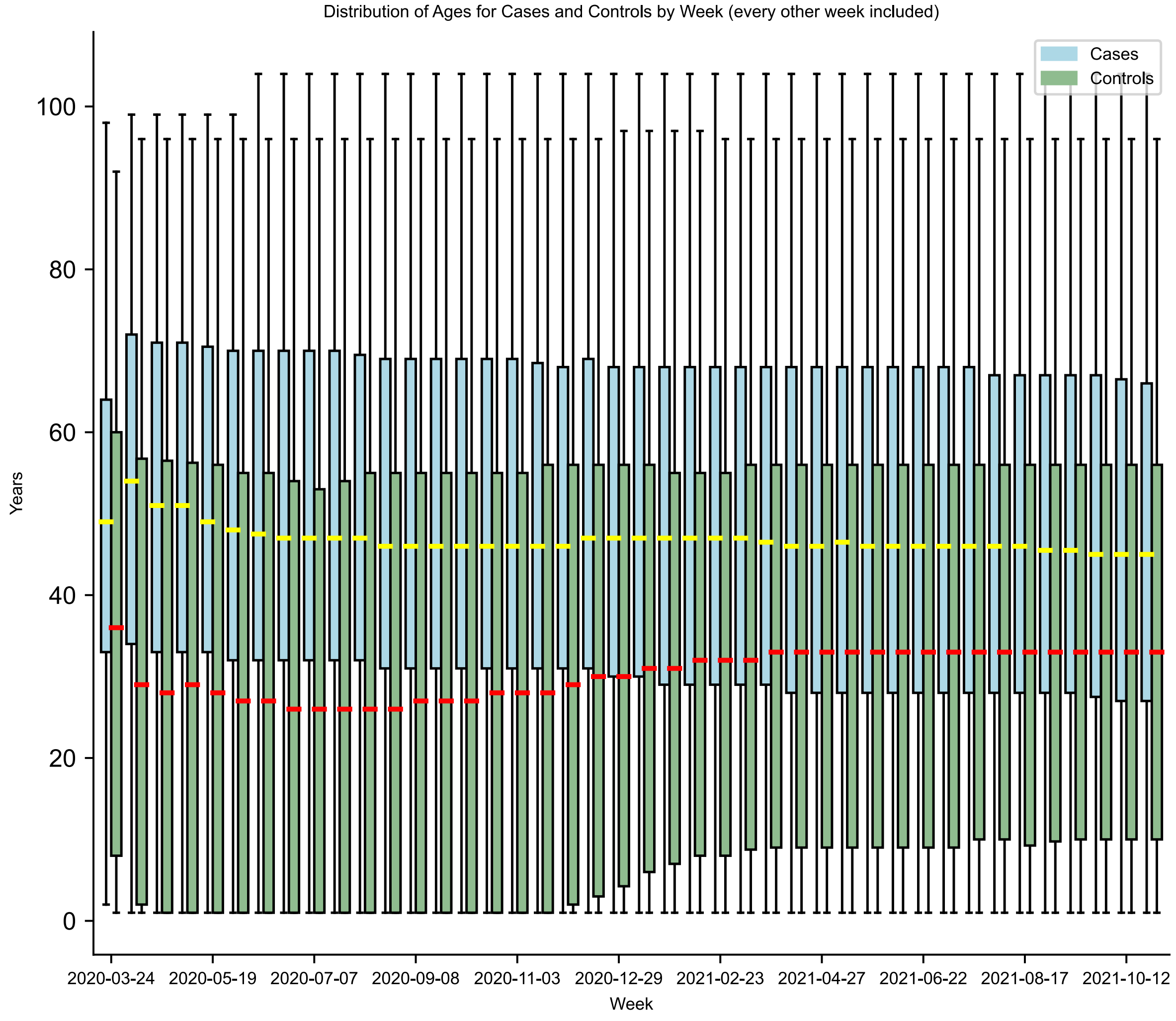

Supplementary Figure 3. Box and whisker plots of ages of case and control groups by week. Every other week excluded for ease of viewing. The center line is the median, the box limits are the first and third quartiles, and the end points are 1.5x the inter-quartile range.

Supplementary Figure 4. Heritability and shared environment estimates over time for SARS-CoV-2 susceptibility with covariates excluded

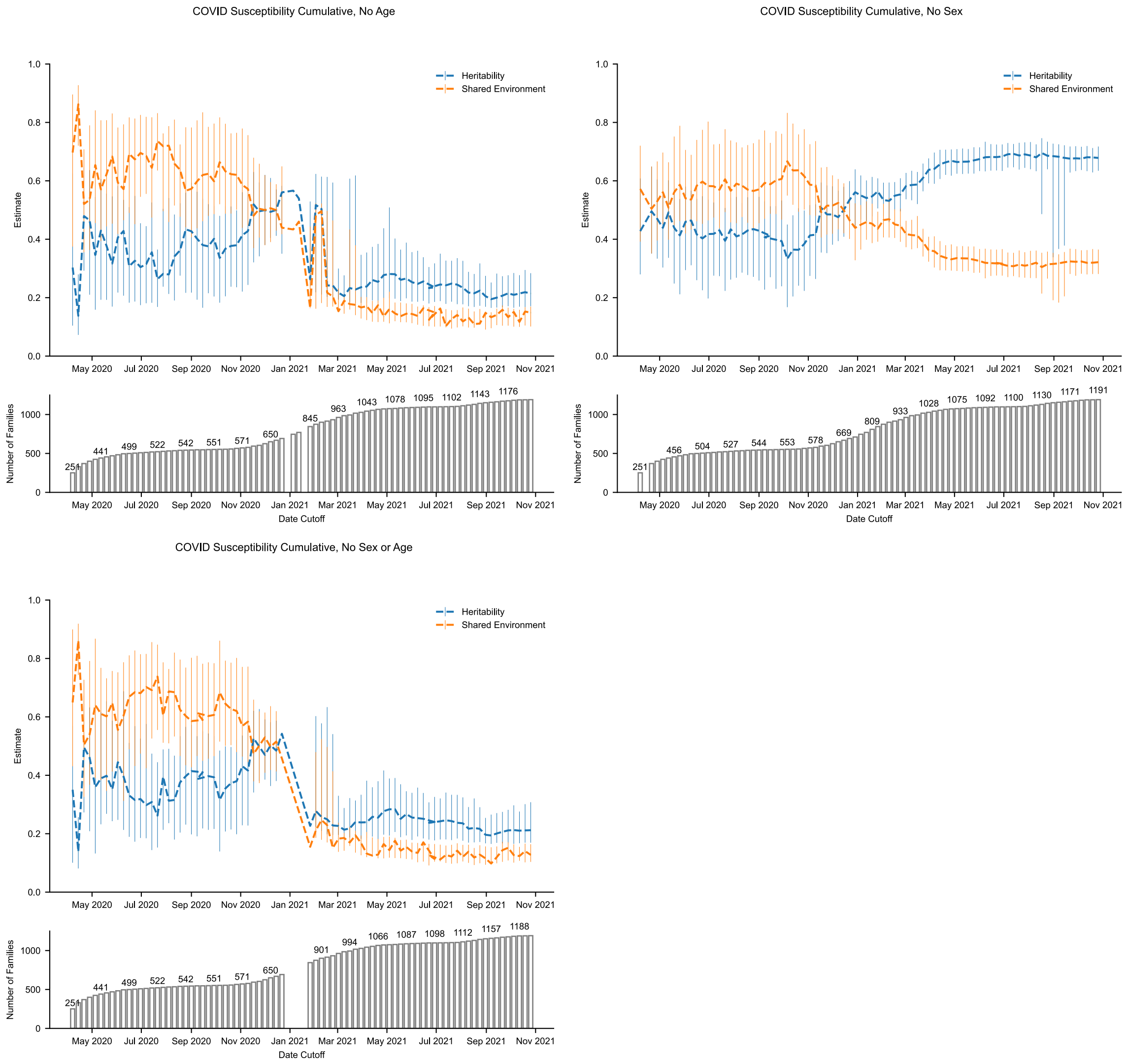

Supplementary Figure 4. Cumulative heritability and shared environment estimates with 95% CI after age, sex, and age and sex covariates removed from models. Bar charts show the number of families in each analysis. Source data are provided as a Source Data file.
