# Supplementary material for: Estimating the heritability of SARS-CoV-2 susceptibility and COVID-19 severity
Source: Nat Commun. 2024 Jan 8;15:367. doi: 10.1038/s41467-023-44250-7 (PMC10774300; doi:10.1038/s41467-023-44250-7)
Supplement: Supplementary file 3 — Reporting Summary [file 41467_2023_44250_MOESM3_ESM.pdf]

nature portfolio

Corresponding author(s): Nicholas Tatonetti

Last updated by author(s): Nov 5, 2023

Reporting Summary

Nature Portfolio wishes to improve the reproducibility of the work that we publish. This form provides structure for consistency and transparency in reporting. For further information on Nature Portfolio policies, see our Editorial Policies and the Editorial Policy Checklist.

Statistics

For all statistical analyses, confirm that the following items are present in the figure legend, table legend, main text, or Methods section.

n/a	Confirmed	
☐	☒	The exact sample size (*n*) for each experimental group/condition, given as a discrete number and unit of measurement
☐	☒	A statement on whether measurements were taken from distinct samples or whether the same sample was measured repeatedly
☐	☒	The statistical test(s) used AND whether they are one- or two-sided<br>*Only common tests should be described solely by name; describe more complex techniques in the Methods section.*
☐	☒	A description of all covariates tested
☐	☒	A description of any assumptions or corrections, such as tests of normality and adjustment for multiple comparisons
☐	☒	A full description of the statistical parameters including central tendency (e.g. means) or other basic estimates (e.g. regression coefficient) AND variation (e.g. standard deviation) or associated estimates of uncertainty (e.g. confidence intervals)
☐	☒	For null hypothesis testing, the test statistic (e.g. *F*, *t*, *r*) with confidence intervals, effect sizes, degrees of freedom and *P* value noted<br>*Give P values as exact values whenever suitable.*
☒	☐	For Bayesian analysis, information on the choice of priors and Markov chain Monte Carlo settings
☒	☐	For hierarchical and complex designs, identification of the appropriate level for tests and full reporting of outcomes
☒	☐	Estimates of effect sizes (e.g. Cohen's *d*, Pearson's *r*), indicating how they were calculated

Our web collection on statistics for biologists contains articles on many of the points above.

Software and code

Policy information about availability of computer code

Data collection	We pulled data from CUIMC/NYP's data warehouse using MySQL version 5.6 and python version 3.8.10. Python scripts and MySQL queries can be found on GitHub at https://github.com/tatonetti-lab/covid-h2o.
Data analysis	We estimated shared environment and heritability estimates using SOLARStrap version 1.0.0 which can be found on GitHub at https://github.com/tatonetti-lab/h2o. Additional analyses were conducted using python version 3.8.10. Scripts for these analyses can be found at https://github.com/tatonetti-lab/covid-h2o.

For manuscripts utilizing custom algorithms or software that are central to the research but not yet described in published literature, software must be made available to editors and reviewers. We strongly encourage code deposition in a community repository (e.g. GitHub). See the Nature Portfolio guidelines for submitting code & software for further information.

Data

Policy information about availability of data

All manuscripts must include a data availability statement. This statement should provide the following information, where applicable:
- Accession codes, unique identifiers, or web links for publicly available datasets
- A description of any restrictions on data availability
- For clinical datasets or third party data, please ensure that the statement adheres to our policy

We use electronic health records which are protected in the United States from public access through the 1996 Public Law 104-191 (HIPAA). Please send all requests for additional materials to the corresponding author who will respond within 2 weeks.

Research involving human participants, their data, or biological material

Policy information about studies with human participants or human data. See also policy information about sex, gender (identity/presentation), and sexual orientation and race, ethnicity and racism.

Reporting on sex and gender	We categorize patient sex as "female" and "not female" in our analysis based on patient provided data, and control for sex as a covariate in our analyses.
Reporting on race, ethnicity, or other socially relevant groupings	We report race/ethnicity sample summary information but do not explicitly include this in our analyses. Race and ethnicity is self-reported. The grouping algorithm for combining race and ethnicity information can be found in the data processing scripts mentioned in the software and code section.
Population characteristics	67% of included patients are female. 20% of patients are Hispanic, 11% are Black, 17% are White, and 51% are Other.
Recruitment	No patients were recruited. This is an observational study based on secondary use of electronic health record data.
Ethics oversight	The study was approved by Columbia University's Institutional Review Board.

Note that full information on the approval of the study protocol must also be provided in the manuscript.

Field-specific reporting

Please select the one below that is the best fit for your research. If you are not sure, read the appropriate sections before making your selection.

☒ Life sciences ☐ Behavioural & social sciences ☐ Ecological, evolutionary & environmental sciences

For a reference copy of the document with all sections, see nature.com/documents/nr-reporting-summary-flat.pdf

Life sciences study design

All studies must disclose on these points even when the disclosure is negative.

Sample size	Overall sample size reported is the number of patients in the most permissive run of SOLARStrap. These are patients that 1) have a conclusive PCR test in NYP/CUIMC data warehouse and 2) is a part of a family in our pedigree with at least one other family member that has a conclusive PCR test (requirement for inclusion in SOLARStrap). For each analysis that includes a subset of these patients, we report the number of families included per analysis since SOLARStrap samples at the family level to determine which patients to include in each iteration.
Data exclusions	Susceptibility controls were excluded if they had ICD code Z86.16 which codes for history of COVID-19.
Replication	We repeated our analyses using between 20% and 90% of available families at 10% increments to see how changing number of families effects the results. The model did not converge on an estimate for the main analyses (requiring a proband and including all covariates) in 3 out of 24 iterations of the model - 20% family inclusion, 30% family inclusion and 90% family inclusion for Hospitalization Status.
Randomization	Randomization is not appropriate for this study since it uses an observational dataset and no intervention is tested.
Blinding	Blinding is not appropriate here since we use an observational dataset and do not test an intervention.

Reporting for specific materials, systems and methods

We require information from authors about some types of materials, experimental systems and methods used in many studies. Here, indicate whether each material, system or method listed is relevant to your study. If you are not sure if a list item applies to your research, read the appropriate section before selecting a response.

Materials & experimental systems

n/a	Involved in the study
☒	☐ Antibodies
☒	☐ Eukaryotic cell lines
☒	☐ Palaeontology and archaeology
☒	☐ Animals and other organisms
☒	☐ Clinical data
☒	☐ Dual use research of concern
☒	☐ Plants

Methods

n/a	Involved in the study
☒	☐ ChIP-seq
☒	☐ Flow cytometry
☒	☐ MRI-based neuroimaging

Plants

Seed stocks

Report on the source of all seed stocks or other plant material used. If applicable, state the seed stock centre and catalogue number. If plant specimens were collected from the field, describe the collection location, date and sampling procedures.

Novel plant genotypes

Describe the methods by which all novel plant genotypes were produced. This includes those generated by transgenic approaches, gene editing, chemical/radiation-based mutagenesis and hybridization. For transgenic lines, describe the transformation method, the number of independent lines analyzed and the generation upon which experiments were performed. For gene-edited lines, describe the editor used, the endogenous sequence targeted for editing, the targeting guide RNA sequence (if applicable) and how the editor was applied.

Authentication

Describe any authentication procedures for each seed stock used or novel genotype generated. Describe any experiments used to assess the effect of a mutation and, where applicable, how potential secondary effects (e.g. second site T-DNA insertions, mosiacism, off-target gene editing) were examined.
